# 

**Published:** 1890-12

**Authors:** 


					LIST OF SUBSCRIBERS
TO
Zbc Bristol flDefcico^Cfotrurgtcal JountaL
DECEMBER, 1890.
BODMIN.
j8nolaitt>.
BERKSHIRE.
MAIDENHEAD.
J. Lidderdale
CHESHIRE.
CHESTER. EGREMONT.
R. E. Burges T. W. A. Napier
CORNWALL.
PADSTOVV. REDRUTH.
W. Pearse H. F. Marley k A. Michdl
CALSTOCK. PENZANCE. ? ST AUSXELL.
H. T. Woodd w. S. Bennett w. Mason
J. A. Fox
LOST WITH IEL. POLRUAN.
J. H. Sewart W. H.Torbock
ST. JUST.
R. B. Searle
NEW QUAY. PORT isaac. j. p. Jackson
A. Hardwick C. Williams. E. Bundle
DEVONSHIRE.
BAMPTON. DAWLISH. HONITON.
T. A. Guinness a. de W. Baker T' w- Sliortridge
F. M. Cann
BARNSTAPLE. LYNTON.
C. H. Gamble devonport. f- c- Berr>'
?rJ-Harper. G. T. Rolston
W. A. G. Laing p ?_ Row modbury.
F- Penny J. H. Harris
BRIXHAM' EXETER- NEWTON ABBOT.
G. C. Searle J. M. Ackland g. Haydon
A. G. Blomfield
H. Davy
broadclyst. p jyp Deas ottery st. mary.
J. Somer E. j. Domville p. a. Gray
A. W. Kempe
CHAGFORD. _ , - PAIGNTON
E. B. Steele-Perkins aiunion.
A. D. Hunt L. H. Tosswill J- Alexander
290 SUBSCRIBERS.
DEVONSHIRE (Continued).
PLYMOUTH. SALCOMBE. TORQUAY
R. H. Clay A. H. Twining
E. E. Meeres VV. Powell
W. G. Nash
E. 13. Thomson south molton. totnes.
W. L. Woollcombe ? Furse ^raser
UFFCULME.
plympton. tavistock. F. Morgan
VV. D. Stamp *p. Leamon winkleigh.
J. H. Norman
ST. MARY CHURCH. TIVERTON. YELVERTON.
T. Finch J. S. Smith A. P. Prowsc
DORSETSHIRE.
BEAMINSTER. PARKSTONE. STURMINSTER NEWTON.
T. P. Daniel W. H. Masters J. C. Leach
F. P. Kitson
POOLE.
BOURTON. J. McNicoll
B. P. Bartlett
WEYMOUTH.
R. VV. Carter
Portland. J- M. Lawrie
G. H. Lillev W- G> V- Lush
DORCHESTER.
F. B. Fisher
E. VV. Kerr sherborne. wimborne.
P. W. MacDonald VV. H. Williams A. J. H. Crespi
ESSEX.
SOUTHEND.
E. E. Phillips
GLOUCESTERSHIRE.
BRISTOL.
VV. R. Ackland E. L. W. Dunbar H. Lawrence
G. Adams VV. Duncan E. L. Lees
J. B. Adams F. H. Edgeworth Museum and Library
G. F. Atchley T. F. Edgeworth VV. E. Lloyd
\V. M. Barclay VV. Elder F. T. B. Logan
T. G. L. Baretti C.Elliott E.J. Maclean
B. J. Baron W. R. Etches W. T. Maddison
J. Beddoe J. Ewens H. Marshall
A. E. Blacker R. G. Fendick C. E. Matthews
A. F. Blagg E. L. Fox T. O. Mayor
E.C. Board W. J. Fyffe J. S. Mettord
J. G. Boyce G. Gardiner J. Milne
J. Broom A. N. G. Gibbs H. F. Mole
G. F. Burder J. Gill C. A. Morton
J. P. Bush G. A. Gloag W. N. Nevill
F. Calder W. C. Goodden C. Newman
J. Cameron W. G. Grace W. H. C. Newnham
A. Carr L. M. Griffiths J. Newstead
W. F. Carter C. D. G. Hailes T. D. Nicholson
J. M. Clarke A. H. Haines J. A. Norton
E. W. Coathupe A. J. Harrison E. H. Openshaw
R. \V. Coe VV. H. Harsant G. S. Page
T. V. Coker A. G. Hayman G. Parker
T. J. Colman J. C. Heaven J. H. Parry
G. G. Corbould C. K. C. Herapath T. C. Parson
W. Cotton H. E. Hetling G. C. P. Pauli
F. R. Cross H. Hill W. J. Penny
J. Dacre F. F. Hills J. H. Perkins
D. S. Davies General Hospital C. F. Pickering
H. F. Devis G. A. Imlay VV. E. Pountney
H. R. Dew Royal Infirmary A. Prichard
N. C. Dobson F. P. Lansdown A. VV. Prichard
VV. Dowson A. E. A. Lawrence J. E. Prichard
SUBSCRIBERS. 2gi
GLOUCESTERSHIRE (Continued).
Bristol (continued).
A. Pring R. S. Smith R. W. Thomas
A. B. Provvse S. Smith W. J. Tivy
W. B. Roue R. V. Solly H. Waldo
C. K. Rudge G. M. Stansi'eld C. H. Walker
H. T. Rudge C. Steele C. H. Wallace
H. F. Semple W. H. Stevens J. H. Wathen ?
J. E. Shaw F. W. Stoddart T. Webster
E. J. Sheppard J. Swain C. E. Wedmore
W. E. Shorland J. G. Swayne R. R. Whishaw
E. M. Skerritt S. H. Swayne J. Wilding
G. M. Smith W. C. Swayne P. W. Williams
J. G. Smith J. Taylor H. W. Windsor-Aubrey
CHALFORD. HAMBROOK. STOW-ON-THE-WOLD.
F. C. Palmer E. Crossman E. Dening
W. F. B. Eadon
CHELTENHAM. STROUD.
Cheltenham Library hawkesbury-upton. a. S. Cooke
?eivt?n, W. I. Cox E. A. Howsin
F. A. A. Smith w. H. Paine
L. Winterbotham kingswood. F. W. Storry
chipping sodbury. H. Grace
TETBURY.
MORETON-IN-MARSH. Wickham
A. Grace
CINDERFORD. L. K. Yelf
W. C. Heane thornbury.
staple hill. E. M. Grace
coleford. W. Murray T. H. Taylor
L. B. Trotter
downend. h a Benhara
H. Skelton
STAPLETON. WESTI1UR Y-ON-TRYM.
H. Ormerod
WINTERBOURNE.
FISHPONDS. ST0KK BISHOP.
W. Brown H- Appleton R E
J. Stewart i'?n,er
GLOUCESTER.
r> w Rnttpn STONF.HOUSE. WOTTON-UNDER-EDGE.
T. S. Ellis G. T. B.Walters D. H. Forty
HAMPSHIRE.
BEMBRIDGE. CARISBROOKE. ST. MARY BOURNE.
T. D. Pain J. Groves. A. W. Riddell
BOURNEMOUTH. EMSWORTH. SOUTHSEA.
E. F. Bright L. Stephens J- W. Cousins
D. C. Embleton vfn'txor
J. Horsfall lymington. \entmok.
S. T. Smyth J. Rendall R- Robertson
HEREFORDSHIRE.
HEREFORD.
W. C. Whitfeld
HERTFORDSHIRE.
ST. ALBANS.
A. H. Boys
KENT.
DARTFORD. TUNBRIDGE WELLS.
A. H. Syree C. Wilson
292
SUBSCRIBERS.
LANCASHIRE.
LIVERPOOL. POULTON-LE-FYLDE.
Medical Institution P.H.Day
MIDDLESEX.
ISLEWORTH.
W. A. Moynan.
LONDON.
J. Abercombie M. Grieor , W. Pippette
I S Beale M. Handfield-Jones R. D. Powell
I Browne J- Hutchinson M. A. Ruffer
T Buzzard W. H. A. Jacobson M. W. H. Russell
J G Davey C. M. Jessop VVJ. Seward
I F Evans J- Lister N. Smith
A P Gould W. C.Luffman Royal Med. and Chir. Society
J. M. Granville J. Macready J. A. Watson
G. S. Gregory A. L. Marshall F. J. Wethered
TOTTENHAM.
W. H. Plaister
MONMOUTHSHIRE.
NANTYGLO. PONTYPOOL. RHYMNEV ?
W, Bevan S. 13. Mason T. H. Redwood
NEWPORT. RISCA.
W. Basset A. G. Thomas G. B. Robathan
H. R. Hudson ]? T. Thomas trfdegar
T. Limbery H. E. Williams tredegar.
O. E. B. Marsh G- A- Brown
NORTHAMPTONSHIRE.
PETERBOROUGH.
T. Edwardes
NOTTINGHAMSHIRE.
NOTTINGHAM.
C. B. Taylor
OXFORDSHIRE.
OXFORD.
R. W. Doyne
SOMERSETSHIRE.
BANWELL.
A. J. Bisdee
BATH.
H. T. S. Aveline W.M.Ellis L.J.King
G. E. Bloxam A. E. W. Fox F. Lace
A. B. Brabazon H. W. Freeman H. Lane
C. Coates H. F. A. Goodridge T. P. Lowe
T. Cole T. B. Goss R. J- H. Scott
F S. Cowan F. K. Green E. Skeate
S Craddock F. P. Hoblyn J- K. Spender
r. Davis H. C. Hopkins L. A. Weatlierly
BECKINGTON. BRISLINGTON. CHEW MAGNA.
W. G. Evans 13. u. pox E- T' Hale
C. H. I'OX CLEVEDON.
BRENT KNOLL. T Davis
J. W. Papillon burnham. g. F. P. Pizey
A. W. C. Peskett S. Skinner
BRIDGWATER.
CREWKERNIi.
T. L. Laxton CHEDDAR. _
Fj . ^Siifcock S R- W. Statham W. W. Webber
SUBSCRIBERS. 293
SOMERSETSHIRE (Continued).
DUI.VERTON. MINEHEAD. WEDMORE.
R. J. Collyns T. Clark J- Ford
G. F. Sydenham T. J. Ollerliead R- P- Tyley
FRESHFORD. NORTH PETHERTON. WELLINGTON.
C. E. S. Flemming C.F.Hawkins J.Meredith
FROME. PILL. T- E- P- PlideaUX
E. Cockey R. A. Ross wells.
rF;rP? portishead. W. Fairbanks
J'M-RaUra> W. Monckton A. L. Wade
GLASTONBURY. C. A. Wigan WESTON-SUPER-MARE.
J. A. Bright south petherton. G. E. Alford
huntspill. W. H. Walter E. F. H. Burroughs
t tt G. B. Fraser
J. H. Sharpe stogumber. g p_ Martin
C. E. S. Brettingham W. H. G. Phelps
1LMINSTER.
C. Munden street
G. F. Rossiter
R. Roxburgh
A. Clarke F. W. S. Wicksteed
KEYNSHAM.
G. G. D. Willett taunton. wrington.
H. Alford a T-T npnqfin
langport. h. J. Alford H. W. Collins
J. iMorgan J- Carey
Taunton Hospital yatton.
midsomer NORTON W. Liddon A de c Lyons
P'aC^?r TEMPLE CLOUD.
A. Waugh ? YEOVIL.
T. Martin
MILVERTON. TWERTON-ON-AVON
P. S. H. Colmer
C. J. Marsh
C. Randolph J. Wigmore E. Vernon
STAFFORDSHIRE.
LONGTON. STAFFORD.
E. D. Duffett J. Scott
SURREY.
esher.
J. B. Fry
SUSSEX.
EASTBOURNE. . ST. LEONARD'S-ON-SEA.
W. B. Beatson W. H. Spencer
WARWICKSHIRE.
BIRMINGHAM.
L. Tait
WILTSHIRE.
BURBAGE. FOVANT. STRA'TTON ST. MARGARET.
A. E. N. Browne C. Clay J. Fernie
CALNE.
MALMESBURY. SWINDON.
R. Kinneir W. Howse
W-S-Batte? -X" J. C. Maclean
SALISBURY.
DEVJZES. H WARMINSTER.
T. B. Anstie L. S. Luckhani J- Hinton
C. Eddowes H. J. Manning R- L. Willcox
WORCESTERSHIRE.
WORCESTER.
J. F. Royle
YORKSHIRE.
HARROGATE. SHEFFIELD. WAKEFIELD. YORK.
A. O. Jones S. Snell A. R. Steel L. H. Williams
294 SUBSCRIBERS.
3relanfc.
ANTRIM. LOUTH
BELFAST. LOUTH.
J. W. Browne J. Browne
Scotland
EDINBURGHSHIRE.
EDINBURGH.
Y. J. Pentland
Males.
BRECONSHIRE.
BRECON. BRYNMAWR. SENNY BRIDGE. TALGARTH.
A. Whyte G. H. Browne W. R. Jones W. Howells
CARDIGANSHIRE.
LAMPETER. NEW QUAY.
E. C. Davies E. R. Jones
CARMARTHENSHIRE.
CARMARTHEN. FERRYSIDE. LLANELLY.
A. Devonald P. Williams D. J. Williams
G. J. Hearder
GLAMORGANSHIRE.
ABERAMAN. ABERAVON. ABERDARE. CAERPHILLY.
J.B.James J.A.Jones E.Jones J. Lewellyn
CARDIFF.
W. T. Edwards D. R. Paterson Medical Society
G. W. Grote W. Price J. T. Thompson
J. Milward R. Prichard C. T. Vachell
W. Morgan A. Sheen H. R. Vachell
LLANDAFF. MERTHYR TYDVIL.
J. Arthur J. L. W. Ward
PENARTH. PORTH. REYNOLDSTONE.
W. J. Clapp H.N. Davies H. V. Ellis
SWANSEA. TUEHARRIS.
E. Davies J. A. Rawlings W. W. Leigh
G. Padley J. Thomas
PEMBROKESHIRE.
TENBY.
B. Kendall
Channel 3slanfcs. 3sle of Man.
GUERNSEV. RAMSEY.
N. Webster H. H. Tomkins
Greece. 3tal\>. Swutserlanfc.
CORFU. PALERMO. DAVOS-PLATZ.
Dr. Mavrojanni C. Scimonelli W. R. Huggard
HlGeria.
ALGIERS.
\V. Thomson
INDEX.
Abdomen, Surgical physiology
of?T. S. Ellis, 98.
Actinomycosis (Abstract), 56.
Alford, H. ? The Bristol In-
firmary, 165.
American resorts?B. W. James
(Review), 44. '
Ammonium chloride inhalation,
Instrument for, 46.
Aneurism, orbital, Ligature of
carotid ? A. W. Prichard,
II5-
Angina, from affection of vagus
(Abstract), 55.
Angina pectoris, Varieties (Ab-
stract), 137.
Annual, The medical (Rcvieiv),
45-
Annual of Universal Med. Sc.,
1890 (Review), 202.
Antipyrin (Abstract), 68.
Antisepsis, Intestinal (Abstract),
212.
Ataxia locomotor, Treatment
(Abstract), G8.
Australian med. congress trans.
(Review), 132.
Baron, B. J.?Outgrowths on
tongue, 80.
Beaumont, W. M.?The shadow-
test (Review), 274.
Binder, The (Abstract), 286.
Blomfield, A. G. ? Case of
Landry's paralysis, 113.
Braithwaite's retrospect of medi-
cine (Revietv), 45.
Bramwell, Byrom?Studies in
clinical medicine [Review), 44.
Bromoform in whooping cough
{Abstract), 285.
Bronchitis (chronic) and its
treatment?W. Murrell (Re-
vieiv), 43.
Broncho-pneumonia, Erysipela-
tous [Abstract), 137; Diagnosis
in children [Abstract), 138.
Caffyn's liquor carnis, 277.
Children, Disease in?A. Money
(Review), 201.
Children's diseases, Clinical
studies?E. L. W. Dunbar, 192.
Children's deformities?YV. Pye
[Review), 132.
Chloralamide [Abstract), 143.
Chloroform syncope?R. Kirk
[Revieiv), 203.
Clarke, J. M.?Paraplegia, 226.
Clevenger, S. N.?Spinal con-
cussion [Review), 133.
Cocoa essence, 207.
Cod-liver oil, Alkaloids [Ab-
stract), 142.
Concato's disease (Abstract), 211.
Congress, International medical
[Notice), 49.
Cox, F. A.?The care of the
skin (Revieiv), 205.
Cripps, H,?Diseases of the
rectum and anus (Review), 41.
Crookshank, E. M. ? History
and pathology of vaccination
(Review), 129.
2g6 INDEX.
Denaeyer's peptones, 47.
Dental surgery ? H. Sewill
{Review), 205.
Diagnosis, Clinical ? v. Jaksch
(Review), 199.
Diagnosis, Physical, Handbook
?Dr. C. J. Nixon (Review), 38.
Dressings, Surgical(/4 bstract), 282.
Duckworth, Sir D.?Treatise on
gout (Review), 36.
Dunbar, E. L. W. ? Clinical
studies, 192.
Edgeworth, Dr. F. H.?Sequelae
of otitis media, 18, 87.
Education committee report
(Review), 268.
Electricity in treatment of uterine
tumours ? T. and S. Keith
(Review), 39.
Ellis, T. S.?Surgical physiology
of abdomen, 95.
Epistaxis premonitory of ne-
phritis (Abstract), 55.
Erysipelas, Treatment (Abstract),
66.
Exostoses, Multiple ? E. D.
Maclean, 217.
Exalgine (Abstract), 62, 206.
Food in health and disease?
I. Burney Yeo (Review), 43.
Fothergill, J. M. ? The town
dweller (Review), 130.
Frame food preparations, 275.
Fyffe, W. J. ? Epidemic in-
fluenza, 147.
Gall-stones,Treatment (Abstract),
64.
Gant, F. J. ? The student's
surgery (Review), 40.
Gastric juice influencing germs?
(Abstract), 57.
Genital canal, Disinfection of
(Abstract), 67.
Goitre, exophthalmic, Treatment
(Abstract), 63.
Gout, Treatise on?Sir D. Duck-
worth (Review), 36.
Harrison, Dr. A. J.?Factitious
urticaria, 32.
Health, Wanderings in search of
?H. C. Taylor (Revieiv), 131.
Heart-disease, Congenital ? L.
Stephens, 196.
Hernia, Radical cure of ?J.
Greig Smith, 1.
Hutchinson, J. ? Archives of
surgery (Review), 202.
Hygiene?L. C. Parkes (Review),
203.
Ilfracombe?E. J. Slade-King,
257-
Infirmary, The Bristol ? H.
Alford, 165.
Influenza, Epidemic ? W. J.
Fyffe, 147.
Insomnia?A. W. Macfarlane
(Review), 131.
Intestinal antisepsis (Abstract),
212.
Isinglass, Patent refined, 276.
Jaksch, v. ? Clinical diagnosis
(Review), 201.
Keith, T. & S.?Treatment of
uterine tumours by electricity
(Review), 39.
Kirk, R.?Chloroform syncope
(Revieiv) 203.
Kolatina, 135.
Lanoline toilet, 135.
Laryngeal tuberculosis, Sulphur-
ous water in (Abstract), 62.
Larynx, epithelioma, treatment
(Abstract), 63.
Lawrence, A. E. A. ? Post-
partum complications, 73.
Leprosy and its prevention ?
Robson Roose (Review), 42.
Macfarlane, A. W.?Insomnia
(Review), 131.
Maclean, E. J.?Multiple exos-
toses, 217.
Malt extract, The standard, 207.
Medicine, Studies in clinical?
Byrom Bramwell (Revieiv), 44.
Mercury during Albuminuria
(Abstract), 214.
Money, A.?Disease in children
(Review), 201.
Morvan's disease (Abstract), 139.
Murrell, W.?Chronic bronchitis
and its treatment (Review), 43.
INDEX. 2Q7
Nervous diseases?A. L. Ranney
(Review), 37.
Neuritis, peripheral ? D. R.
Paterson, 244.
Nixon, C. I.?Physical diagnosis
(Review), 38.
Nona (Abstract), 141.
Obstetric nursing, Handbook of
(Review)?Haultain and Fer-
guson, 126.
(Jrexin (Abstract), 143.
Organisms in air (Abstract), 57.
Orthin (Abstract), 65.
Otitis media, sequelae?Dr. F.
H. Edgewortli, 18, 87.
Ouabin liquor, 134.
Owen, E.?Surgery of infancy
and childhood (Review), 273.
Paralysis (Landry's),case?A. G.
Blomfield, 113.
Paraplegia, Varieties ?J. M.
Clarke, 226.
Parkes, L. C.?Hygiene (Review),
203.
Paterson, D. R. ? Peripheral
neuritis, 244.
Peptones, Denaeyer's, 47.
Pharmacopoeia, The extra
(Review) 126.
L'henyl urethan (Abstract), 68.
Phthisis, Pulmonary, creasote in
(Abstract), 60.
Physiognomy ? M. O. Stanton
(Review), 273.
Pills, Gelatin-coated, 49.
Pneumonia, Fibrinous, infectious
(Abstract), 55.
Pneumonia after injury (Abstract),
281.
Podophyllin, New preparations
of, 48.
Polyorromenitis (Abstract), 211.
Post-partum complications ? A.
E. A. Lawrence, 73.
Prichard, A. W.?Orbital aneu-
rism, ligature of carotid, 115.
Pulse, Centripetal venous
(Abstract), 141.
Pye, W.?Children's deformities
(Review), 132.
Pyoktanin (Abstract), 143.
Ranney, A. L. ? Nervous dis-
eases (Review), 57.
Rectum and anus, Diseases of
?H. Cripps (Review), 41.
Rheumatic diseases ? H. Lane
and C. J. Griffiths (Review),
12 7.
Rigaud and Chapteaut ? New
remedies, 47.
Roose, Robson?Leprosy and its
prevention (Review), 42.
Salicylate of soda (Abstract), G4.
Salol (Abstract), 6S, 284.
Salts, Wiesbaden, 277.
Salt Regal, 135.
,, Sprudel, 277.
,, Franz Joseph, 277.
Sewill, H. ? Dental surgery
(Review), 205.
Shadow-test, The?W. M. Beau-
mont (Review), 274.
Skin, The care of the?F. A.
Cox (Review), 205.
Slade-King?Ilfracombe, 257.
Smith, J. Greig ? Radical cure
of hernia, 1.
Specific and non-specific diseases
(Abstract), 139.
Spinal concussion?-S. N. Clev-
enger (Review), 133.
Spinal cord, Surgery of?W.
Thorburn (Review), 132.
Stephens, L.?Congenital heart-
disease, 196.
Stanton, M. O.?Physiognomy
(Review), 273.
Strychnine in cerebral affections
(Abstract), 285.
Sulphur in fumigation (Abstract),
7?-
Surgery, Archives of?J. Hutch-
inson (Review), 202.
Surgery, Dental ? H. Sewill
(Review), 205.
Surgery of infancy and child-
hood?E. Owen (Review), 273.
Surgery, The student's?F. J.
Gant (Review), 40.
Surgical pathology?W. J. Wals-
ham (Review), 42.
Swayne, J. G.?Epithelioma of
uterus, 119.
Sweating feet, treatment (Ab-
stract), G5.
Taylor, H. C.?Wanderings in
search of health (Review), 131.
298 INDEX.
Theobromin (Abstract), 66.
Thorburn, W.?Surgery of spinal
cord (Review), 132.
Throat, New remedies in diseases
of (Abstract), 64.
Thyroid, Tetany after extirpa-
tion of (Abstract), 54.
Tongue-outgrowths?B.J. Baron,
80.
Town dweller, The ? J. M.
Fothergill (Review), 130.
Transactions Australian medical
congress (Review), 132.
Tuberculosis, Experimental (Ab-
stract), 278.
Tuberculosis, Pulmonary, recent
treatment (Abstract), 58.
Urethan phenyl (Abstract), 68.
Urticaria, Factitious, case ? A.
J. Harrison, 32.
Urticaria, Factitious, case ? H.
Waldo, 29.
Uterine tumours, Electricity in
?T. and S. Keith (Review), 39.
Uterus, Epithelioma of body of
?J. G. Swayne, 119.
Vaccination, History and path-
ology of?E. M. Crookshank
(Review), 129.
Varices of (Esophagus (Abstract),
,279;
Vinolia soap, 136.
Vomiting of pregnancy (Abstract),
65-
Waldo, H.?Case of factitious
urticaria, 29.
Walsham, W. J.?Handbook of
surgical pathology (Review), 42.
Year-book of treatment (Review),
45-
Yeo, J. Burney?Food in health
and disease (Review), 43.
J. W. Anowsinith, Printer, Stieut, Uiibtol.
i
Cases for binding, fvicc yd. cach, may be had of the Publisher,
J. W. Arrowsmith, Quay Street, Bristol.
PUBLICATIONS RECEIVED.
From Bailliere, Tindall & Cox, London :
The Medical Press and Circular. September 17th, November 19th, December
5th, 1890.
Diseases of the Skin ami Hair. By R. Glasgow Patteson. 1S91.
The Hospital Gazette. September 20th, 1890.
Urinary Disorders. By Reginald Harrison. 1890.
From 24 Bouverie Street, London :
The Newsman. No. 1. October, 1890.
From The Bristol Urban Sanitary Authority :
The Bristol Mercury. November 22nd, 1890.
From Burroughs, Wellcome & Co.:
The ABC Medical Diary and Visiting List. 1891.
From Ev. E. Carreras, St. Louis:
The Alienist and Neurologist. October, 1890.
From J. & A. Churchill, London:
Differentiation in Rheumatic Diseases (so called). By Hugh Lane. 1890.
Handbook to Dr. Koch's Treatment of Tubercular Disease. By Edward F. Grun
and Walter D. Severn. [November 29th, 1890.]
From F. A. Davis, Philadelphia :
Ointments and Oleates. By John V. Shoemaker, M.D. Second Edition. 1S90.
Epilepsy. By Hobart Amory Hare, M.D. 1890.
Text-Book of Hygiene. By George H. Rohe, M.D. Second Edition. 1890.
The Medical Bulletin Visiting List. 1891.
Diabetes. By Charles W. Purdy, M.D. 1S90.
Electricity in Diseases of Women. By G. Betton Massey, M.D. Second
Edition. 1890.
Heredity, Health, and Personal Beauty. By John V. Shoemaker, M.D. 1890.
Structure of the Central Nervous System. By Dr. Ludwig Edinger. 1890.
Principles of Surgery. By N. Senn, M.D. 1890.
From Wm. J. Dornan, Philadelphia :
On the Treatment of Eczema in Elderly People. By L. Duncam Bulkley, M.D.
1890. Reprint.
From Feret, Bordeaux:
Annates de la Polyclinique. No. 4. July, 1890.
From 330 & 332 Gray's Inn Road, London ;
Pharmacopoeia of Central London Throat, Nose, and Ear Hospital. Compiled by
The Honorary Medical Staff. 1890.
From Charles Griffin & Company, London:
Foods and Dietaries. By R. W. Burnet, M.D. 1890.
From H. K. Lewis, London:
Should Hypnotism have a Recognised Place in Ordinary Therapeutics ? By
Norman Kerr, M.D. 1890.
The British Journal of Dermatology. October?December, 1890.
Sterility in Women. By Arthur W. Edis, M.D. 1890.
Diseases of the Ear. By Dr. Josef Gruber. 1890.
Theory and Practice of Medicine. By Frederick T. Roberts, M.D. Eighth
Edition. 1890.
Diseases of the Eye. By Henry A. Swanzy, M.B. Third Edition. 1890.
Typhoid Fever and Tropical Life.. By Jeffery A. Marston, M.D., C.B. 1890.
Lewis's Nursing Charts.
From Longmans, Green & Co., London:
The Treatment of Syphilis of the Nervous System. By Julius Althaus, M.D.
1890.
From The Open Court Publishing Co., Chicago:
The Monist. Vol I. No. 1. October, 1890.
From Young J. Pentland, Edinburgh :
The Feeding and Care of Infants. By John Service. 1890.
Technic of Manual Treatment. By Arvid Ivellgren, M.D. 1890.
From John Morgan Richards, London:
Medical Reprints. Nos. 9, 10. 1S90.

				

